# Die laienverständliche Version der 2018 EULAR Empfehlungen zu körperlicher Aktivität von Menschen mit entzündlich-rheumatischen und degenerativen Erkrankungen: Übersetzung ins Deutsche und sprachliche Validierung im deutschsprachigen Raum mit Betroffenen

**DOI:** 10.1007/s00393-021-01079-z

**Published:** 2021-10-07

**Authors:** K. Niedermann, A. K. Rausch, J. Braun, H. Becker, P. Böhm, R. Bräm, G. Gilliam-Feld, D. Kiefer, R. Kurz, M. Schönfelder, T. Stamm, U. Kiltz

**Affiliations:** 1 Department Gesundheit, Institut für Physiotherapie, Zürcher Hochschule für Angewandte Wissenschaften ZHAW, Katharina-Sulzer-Platz 9, 8401 Winterthur, Schweiz; 2 Departement Gesundheit, Institut für Physiotherapie, Zürcher Hochschule für Angewandte Wissenschaften, Winterthur, Schweiz; 3 Rheumazentrum Ruhrgebiet, Ruhr-Universität Bochum, Herne, Deutschland; 4 Schweizerische Polyarthritiker Vereinigung, Zürich, Schweiz; 5 Deutsche Vereinigung M. Bechterew, Schweinfurt, Deutschland; 6 Schweizerische Vereinigung M. Bechterew, Zürich, Schweiz; 7 Rheuma-Liga Nord-Rhein-Westfalen, Deutsche Rheumaliga, Essen, Deutschland; 8 Österreichische Vereinigung Morbus Bechterew, Wien, Österreich; 9 Österreichische Rheumaliga, Maria Alm, Österreich; 10 Institut für Outcomes Research, Medizinische Universität Wien, Wien, Österreich

**Keywords:** Bewegungsempfehlungen, Training, Arthrose, Arthritis, Patientenorganisation, Physical activitiy recommendations, Training, Osteoarthritis, Arthritis, Patient organisation

## Abstract

**Hintergrund:**

Körperliche Aktivität und spezifisches Training haben großen gesundheitlichen Nutzen. Empfehlungen zum Management von rheumatoider Arthritis (RA), Spondyloarthritis (SpA) sowie Hüft- und Kniegelenkarthose (HKA) sind bisher in Bezug auf Art und Dosierung aber unspezifisch. Darum wurden die 2018 EULAR Empfehlungen zu körperlicher Aktivität für Menschen mit entzündlich-rheumatischen und degenerativen Erkrankungen formuliert. Sie bestehen aus 4 übergeordneten Prinzipien und 10 Empfehlungen. Die EULAR Bewegungsempfehlungen wurden auch als laienverständliche Version in englischer Sprache publiziert.

**Ziel:**

Übersetzung der laienverständlichen Version ins Deutsche und sprachliche Validierung in Deutschland, Österreich und der Schweiz.

**Methoden:**

Eine professionelle Übersetzung ins Deutsche wurde von den Autor*innen einschließlich Personen mit RA, SpA und HKA zu einer präfinalen Version bearbeitet. Anschließend wurden in den 3 Ländern je 8 Interviews mit Personen mit RA, SpA und HKA durchgeführt, um die Verständlichkeit, Wortwahl, Vollständigkeit und Umsetzbarkeit zu prüfen. Die Patientenvertreter*innen der Autor*innengruppe bewerteten anonym ihre Zustimmung zur finalen Version auf einer Skala von 0 bis 10.

**Ergebnisse:**

Die professionelle Übersetzung wurde von den Autor*innen und auf Grundlage der Interviews substanziell überarbeitet. Dabei wurden Formulierungen angepasst, um Lesbarkeit und Verständlichkeit zu verbessern und Aussagen zu präzisieren. Inhalt und Struktur des Originaltextes wurden dabei nicht verändert. Die Zustimmung zu den einzelnen Empfehlungen lag zwischen 10 (SD 0) und 7,6 (SD 1,67).

**Diskussion:**

Für Menschen mit RA/SpA/HKA sollten die EULAR Bewegungsempfehlungen in deren Muttersprache vorliegen. Die laienverständliche deutsche Version ist valide und wurde in allen 3 Ländern gut akzeptiert. Damit können die EULAR Bewegungsempfehlungen verständlich und praktikabel vermittelt werden.

## Abstract (en)

**Background:**

Physical activity and exercise are beneficial for people with rheumatic diseases; however, recommendations for the management of rheumatoid arthritis (RA), spondyloarthritis (SpA) and hip- and knee osteoarthritis (HOA/KOA) are usually unspecific with respect to mode and dose of exercise. This is why the 2018 EULAR recommendations for physical activity in people with inflammatory arthritis and osteoarthritis were formulated. The recommendations consist of 4 overarching principles and 10 recommendations. These were also published as a lay version in the English language.

**Aim:**

Translation of the lay version into German and its linguistic validation in Austria, Germany and Switzerland.

**Methods:**

A professional translation was reviewed by the authors, including people with, RA, SpA, HOA/KOA from the three German-speaking countries, which provided a prefinal lay version. Subsequently, eight interviews with people with RA, SpA, HOA/KOA were conducted in each country to evaluate understandability, wording, completeness and feasibility of the prefinal lay version. Finally, the authors, i.e. those with RA, SpA, and osteoarthritis, anonymously rated their agreement to the final lay version on a 0–10 scale.

**Results:**

The professional translation was substantially revised by the authors and based on the interviews. Formulations were adapted to increase readability and understandability and specify statements. Comments that would have changed content or structure were not considered. Average agreement with the particular recommendations was between 10 (SD 0) and 7.6 (SD 1.67).

**Discussion:**

For people with RA/SpA/HOA/KOA the EULAR physical activity recommendations should be available in their mother language. The final German lay version is valid and accepted across all three German-speaking countries. Thus, the physical activity recommendations can be provided to people with rheumatic diseases in an understandable and feasible way.

## Hintergrund und Fragestellung

Die laienverständliche Version der 2018 EULAR Empfehlungen zu körperlicher Aktivität von Menschen mit entzündlich-rheumatischen und degenerativen Erkrankungen wurde 2020 ins Deutsche übersetzt und in Deutschland, Österreich und der Schweiz mit Menschen mit rheumatoider Arthritis (RA), Spondyloarthritis (SpA, einschließlich Morbus Bechterew bzw. ankylosierender Spondylitis [axSpA]) sowie Hüft- und Kniegelenkarthrose (HKA) validiert. Die laienverständlichen Empfehlungen sind nun in allen deutschsprachigen Ländern anwendbar und sollen Betroffenen und Patientenorganisationen Orientierung bieten.

Körperliche Aktivität und spezifisches Training wird Gesunden und Menschen mit chronischen Erkrankungen wegen der allgemeinen und krankheitsspezifischen positiven Gesundheitseffekte empfohlen. Körperliche Aktivität ist definiert als jegliche körperliche Bewegung durch die Skelettmuskulatur, die einen Verbrauch von Energie zur Folge hat, z. B. im Haushalt, während der Arbeit oder Fortbewegung [1]. Training ist ein Teilbereich von körperlicher Aktivität, der geplant, strukturiert und regelmäßig durchgeführt wird und das Ziel hat, körperliche Fitness in den Bereichen Herz-Kreislauf (Ausdauer), Muskelkraft, Beweglichkeit und Neuromotorik (Koordination, Gleichgewicht oder Gangsicherheit) zu verbessern oder zu erhalten [2].

Auch für Menschen mit rheumatischen Erkrankungen hat körperliche Aktivität großen Nutzen für Gesundheit und Lebensqualität. Menschen mit entzündlich-rheumatischen Erkrankungen profitieren insbesondere von körperlicher Aktivität in Bezug auf die Krankheitsaktivität [3], während bei HKA Kraft- und neuromotorisches Training eine wirksame Schmerztherapie darstellen [4]. Allerdings sind 50 % der Menschen mit rheumatischen Erkrankungen nicht ausreichend körperlich aktiv – und zwar nicht häufig genug bzw. zu wenig intensiv [5, 6]. Mögliche Gründe dafür sind mangelndes Bewusstsein für den gesundheitlichen Nutzen von körperlicher Aktivität oder Bedenken, dass körperliche Aktivität Krankheitsaktivität oder Gelenkschäden fördern könnte. Krankheitsspezifische Leitlinien und Empfehlungen für rheumatische Erkrankungen betonen fast immer auch die Notwendigkeit körperlicher Aktivität, lassen aber offen, wie oft und wie intensiv trainiert werden soll [7, 8]. Die „2018 EULAR Empfehlungen zu körperlicher Aktivität von Menschen mit entzündlich-rheumatischen und degenerativen Erkrankungen“ (im Folgenden „EULAR Bewegungsempfehlungen“ genannt) wurden mit dem Ziel entwickelt, den im Gesundheitsbereich tätigen Fachpersonen eine diesbezüglich bessere Orientierung zu bieten [9]. Die EULAR Bewegungsempfehlungen sind ein Meilenstein für die Anerkennung des Nutzens von körperlicher Aktivität im Management rheumatischer Erkrankungen. Die EULAR Bewegungsempfehlungen wurden auch in laienverständlicher englischer Sprache publiziert, um die Menschen mit rheumatischen Erkrankungen direkt und verständlich über die Wichtigkeit von körperlicher Aktivität zu informieren (www.EULAR.org).

Die Empfehlungen zu körperlicher Aktivität gelten für Menschen mit RA, SpA und HKA. Sie bestehen aus 4 übergeordneten Prinzipien und 10 Empfehlungen. Eine internationale Expertengruppe aus verschiedenen Ländern und Professionen hatte die Prinzipien und Empfehlungen 2018 formuliert [9]. Sie basieren auf einer systematischen Literaturauswertung von 96 Studien, welche die Einschlusskriterien aus insgesamt 3471 Studien zum Thema körperliche Aktivität bei den gewählten rheumatischen Krankheitsbildern erfüllten. Mit den Wirksamkeitsstudien (RCTs) wurde zudem eine Metaanalyse durchgeführt [10].

Ziele der Studie waren die Übersetzung der laienverständlichen Version der EULAR Bewegungsempfehlungen ins Deutsche und deren sprachliche Validierung in Deutschland, Österreich und der Schweiz. In einem parallelen Projekt wurde auch die Fachversion der EULAR Bewegungsempfehlungen in Deutsch übersetzt und linguistisch validiert.

## Studiendesign und Untersuchungsmethoden

### Übersetzungsprozess

Der Übersetzungs- und Validierungsprozess wurde gemäß den Leitlinien für Best-Practice von interkulturellen Umfragen gemacht [11]. Die Steuerungsgruppe (KN, AKR, JB, TS, UK) lud über nationale Patientenorganisationen in Deutschland, Österreich und der Schweiz die Koautor*innen HB, PB, RB, GGF, RK, MS zu einem eintägigen Treffen ein, um die professionelle Übersetzung der Originalversion zu diskutieren. Diese war, wie die englischsprachige Version, in 7 Teile gegliedert: Einleitung/Was wissen wir bereits darüber/Definitionen/Was sagen die Empfehlungen, inklusive den 4 übergeordneten Prinzipien/Aussagekraft/Die 10 Empfehlungen, inklusive erläuterndem Text/Zusammenfassung.

### Validierungsprozess

Mit der bearbeiteten Version wurden in allen 3 Ländern je 8 kognitive Interviews mit je 2 Personen mit RA, SpA und HKA durchgeführt. Bei der Auswahl der Interviewpartner wurde auf eine breite Verteilung in Bezug auf Alter, Geschlecht, Krankheitsdauer und sozioökonomischen Status (Bildungsgrad und Beruf) geachtet. Mit den halb-strukturierten Interviews wurden die oben genannten 7 Abschnitte mit geschlossenen und offenen Fragen evaluiert – und zwar zu Verständlichkeit (Ist die Aussage verständlich?), Wortwahl (Ist die Wortwahl eindeutig? – Falls nein, Vorschläge), Vollständigkeit (Sind alle Aspekte ausreichend gewürdigt?), Umsetzung (Wird verstanden, wie die Empfehlung umgesetzt werden soll?).

### Finalisierungsprozess und Grad der Zustimmung

Die Steuerungsgruppe diskutierte alle Rückmeldungen, machte ggf. Anpassungen und erstellte eine finale Version. Die Vertreter*innen der Patientenorganisationen gaben den Grad ihrer Zustimmung zu den Prinzipien und den Empfehlungen anonym auf einer Skala von 0 (= stimme überhaupt nicht zu) bis 10 (= stimme vollständig zu). Zwei Erinnerungsmails wurden versendet.

## Ergebnisse

### Übersetzungsprozess

Die professionelle Übersetzung hielt sich eng an die eher knapp formulierte englischsprachige Version. Die Gruppe nahm sprachliche Umformulierungen und Präzisierungen vor, um die Aussagen allgemeinverständlicher zu machen. Eine Herausforderung war es, für alle deutschsprachigen Länder akzeptable und verständliche Begriffe und Formulierungen zu finden. Angepasst wurden beispielsweise:

- „Lay version“, professionell übersetzt als „Anwender- und Patientenversion“, wurde zu „laienverständlicher Version“.
- Die Übersetzung „Menschen mit entzündlicher Arthritis und Osteoarthritis“ wurde durchgehend präzisiert als „Menschen mit RA, SpA, Hüft- und Kniegelenkarthrose“.
- Nach längerer Diskussion einigte sich die Autorengruppe darauf, „Health Care Providers“ als „Gesundheitsdienstleister“ zu übersetzen, mit der Idee, darin alle relevanten Fachpersonen aller 3 Länder einzuschließen. Bei den Empfehlungen wurde, wo angebracht, der Begriff „Behandlungsteam“ verwendet.
- Die Übersetzung „Wirksamkeitsstudien“ wurde mit „beweiskräftigen Studien“ ersetzt.

Das ausführliche Protokoll des Bearbeitungsprozesses kann bei der Erstautorin (KN) angefordert werden.

### Validierungsprozess

Die kognitiven Interviews wurden in jedem Land mit je 2 Personen mit RA, SpA, Hüft- und Kniegelenkarthrose durchgeführt. In einem Land fehlten aus organisatorischen Gründen Personen mit Kniegelenkarthrose (Tab. 1).

**Table 1:** 

	Deutschland	Österreich	Schweiz	Total
*Weiblich (Anteil)*	8 (100 %)	6 (75 %)	5 (63 %)	19 (79 %)
*Alter (Jahre, Durchschnitt (SD))*	59,6 (8,0)	69,3 (8,7)	54,8 (12,7)	61,2 (11,4)
*Rheumatoide Arthritis (RA)*	2	2	2	6 (25 %)
*Spondyloarthritis (SpA)*	2	2	2	6 (25 %)
*Hüftgelenkarthrose*	2	4	2	8 (33 %)
*Kniegelenkarthrose*	2	0	2	4 (17 %)
*Krankheitsdauer (Diagnose seit, Jahre, Durchschnitt (SD))*	3,4 (2,8)	4,9 (5,3)	12,6 (16,2)	7,0 (10,4)
*Funktionseinschränkung*				
Gar nicht	0	1	1	2 (8 %)
Etwas	0	7	7	14 (58 %)
Sehr	8	0	0	8 (33 %)
*Ausbildung in Jahren und höchster Abschluss (Anteil)*	8,0 (0,0)	11,5 (1,4)	15,0 (2,7)	11,5 (3,4)
Abgeschlossene Berufslehre	8	5	4	17 (71 %)
(Berufs‑)Matur	0	2	1	3 (13 %)
Fachhochschule	0	0	1	1
Universität	0	0	2	2
Keine Antwort	0	1	0	1
*Berufstätigkeit (Anteil)*				
Vollzeit	1	1	2	4 (17 %)
Teilzeit	1	1	3	5
Arbeitslos	3	0	0	3
Rente	2	5	2	9
Student/in	0	0	1	1
Hausfrau/mann	0	1	0	1
Andere (bitte beschreiben)	Selbstständig	Keine Antwort	0	2
*Wohnsituation*				
Alleine	4	3	0	7 (29 %)
Mit anderen Personen	4	5	8	17 (71 %)

*SD* „standard deviation“/Standardabweichung

Die Verständlichkeit und Wortwahl kommentierten 16 Teilnehmende (67 %), die Vollständigkeit 8 Teilnehmende (33 %) und die Umsetzung 4 Teilnehmende (17 %). Zu Verständlichkeit gab es 15 Kommentare, zu Wortwahl 15 Kommentare, 14 einzelne Wörter wurden beanstandet und 9 Vorschläge gemacht. Zur Vollständigkeit gab es 8 und zur Umsetzung 4 Kommentare, allgemeine Kommentare insgesamt 37.

Das Protokoll aller Rückmeldungen kann bei der Erstautorin (KN) angefordert werden.

### Finalisierungsprozess

Die Steuergruppe diskutierte alle Rückmeldungen aus den Interviews in einer 4‑stündigen Online-Konferenz. Aufgrund der Kommentare wurden redaktionelle Anpassungen wie kürzere Sätze und Streichungen von Wiederholungen gemacht, um die Lesbarkeit und Verständlichkeit zu verbessern und Aussagen zu präzisieren. Im Folgenden wurden die wichtigsten Anpassungen zusammengefasst.

- *Einleitung/Was wissen wir darüber:* Präzisierung von „Allgemeine Empfehlungen zu körperlicher Aktivität“ zu „Empfehlungen zu körperlicher Aktivität für Gesunde“. Die Begriffe „angeleitet“/„nicht angeleitet“ und der Vergleich mit Medikamenten wurden trotz einzelner Rückmeldungen mit Blick auf die englischen Formulierungen belassen.
- *Definitionen: *Präzisierung durch Wiedereinfügung von 2 Wörtern, die im Übersetzungsprozess verloren gegangen waren: *Ein intensives Ausdauertraining erhöht ****auch**** die Herzfrequenz und führt zum Schwitzen. Jede Woche sollten mindestens 150 Minuten mäßig intensives ****oder ****75 Minuten intensives Ausdauertraining oder eine beliebige Kombination davon durchgeführt werden.*
- *Was sagen die Empfehlungen*: Umformulierung, um die 4 Prinzipien hervorzuheben.
- Kommentare, welche inhaltliche Aussagen oder die Struktur des Originaltextes verändert hätten oder über das Thema hinausgingen, wurden nicht berücksichtigt: Bei *Aussagekraft der Empfehlungen *wurde nicht umgesetzt, die Empfehlungen nach der Anzahl Sterne zu priorisieren, weil alle Empfehlungen gleich wichtig sind und die Reihenfolge von der EULAR Expertengruppe von allgemein zu spezifischer so festgelegt worden war.
- Die *Empfehlungen* wurden nicht, wie mehrfach gewünscht, zu konkreten Handlungsanleitungen umformuliert.

### Finale Zustimmung

Siehe Abschnitt „Empfehlungen“ für die durchschnittlichen Grade der Zustimmung zu den Empfehlungen. Insgesamt gaben 5 der 6 Patientenvertreter*innen ihren Grad der Zustimmung (NRS 0–10) zu der finalen Version an. Die Empfehlungen 1 und 2 erhielten die höchste Zustimmung (Durchschnitt 10), während die Empfehlungen 4 (Durchschnitt 8,4), 6 (Durchschnitt 7,6) und 9 (Durchschnitt 8,6) die tiefsten Zustimmungen erhielten (Tab. 2).

**Table 2:** 

**Einleitung** *Es handelt sich hier um die laienverständliche Version der Empfehlungen zu körperlicher Aktivität für Menschen mit entzündlich-rheumatischen und degenerativen Erkrankungen. Die Empfehlungen wurden verfasst von der Europäischen Rheumaliga (European Alliance of Associations for Rheumatology, EULAR). Sie beziehen sich auf Menschen mit rheumatoider Arthritis (RA), Spondyloarthritis (SpA, einschließlich Morbus Bechterew) und Hüft- und Kniegelenkarthose* *Die Originalpublikation kann über die Internetseite der EULAR unter *www.EULAR.org* heruntergeladen werden*		
Die EULAR Bewegungsempfehlungen geben Gesundheitsdienstleistern und Menschen mit rheumatischen Erkrankungen Ratschläge zum bestmöglichen Krankheitsmanagement. Gesundheitsdienstleister umfassen unter anderem Ärzt*innen, Ergotherapeut*innen, Pflegefachpersonen, Physiotherapeut*innen und Psycholog*innen		
Diese EULAR Bewegungsempfehlungen wurden zusammen mit Patient*innen in einer Arbeitsgruppe entwickelt, um die Patientensicht in den Empfehlungen mit zu berücksichtigen		
*Es gibt bereits internationale Empfehlungen zur körperlichen Aktivität für Gesunde, um die Gesundheit zu verbessern. Mit der von EULAR eingesetzten Arbeitsgruppe sollte herausgefunden werden, ob diese Empfehlungen für Gesunde auch für Menschen mit RA, SpA, Hüft- und Kniegelenkarthrose angewendet werden können*		
*Die RA verursacht Entzündungen in verschiedenen Gelenken, z.* *B. in den Fingern, Zehen, Hüften, Schultern oder Ellenbogen. Die SpA verursacht Entzündungen hauptsächlich in der Wirbelsäule. Die Arthrose ist eine degenerative Erkrankung (Abnützung/Verschleiß), welche häufig die Hüft- oder Kniegelenke betrifft. Diese Krankheiten können zu Schmerzen, Müdigkeit und körperlicher Behinderung führen*		
**Was wissen wir bereits darüber** *Obwohl viele Menschen mit RA, SpA, Hüft- und Kniegelenkarthrose an angeleiteten oder nicht angeleiteten Bewegungsprogrammen teilnehmen, erreichen sie häufig die Empfehlungen zu körperlicher Aktivität für Gesunde nicht. Das ist schade, denn angemessene körperliche Aktivität kann zur Linderung der Krankheitssymptome beitragen und hat auch ganz allgemeine gesundheitliche Vorteile. Körperliche Aktivität kann beispielsweise das Risiko für die Entstehung von Herz-Kreislauf-Erkrankungen verringern. Hinsichtlich der Dosis verhält es sich bei körperlicher Aktivität wie bei Medikamenten. Diese müssen in der richtigen Menge und Häufigkeit eingenommen werden. Auch bei körperlicher Aktivität spielen Häufigkeit, Dauer und Intensität eine gleichermaßen wichtige Rolle*		
**Definitionen** *Körperliche Aktivität umfasst jede Form von Bewegung, die mit einem erhöhten Energieverbrauch einhergeht. Dazu gehören Training und Sport sowie Aktivitäten, die zum täglichen Leben gehören, etwa als Teil des Berufs, im Haushalt, in der Freizeit, oder wenn man sich aktiv von einem Ort zum anderen bewegt*		
*Training ist ein Teilbereich von körperlicher Aktivität. Training wird geplant, strukturiert und wiederholt durchgeführt, um die körperliche Fitness zu erhalten oder wiederherzustellen. Die Weltgesundheitsorganisation (WHO) und das American College of Sports Medicine (ACSM) empfehlen, jeden Tag mäßig intensives Ausdauertraining durchzuführen. Mäßig intensives Ausdauertraining ist zügiges Gehen, Treppensteigen, Radfahren oder Gartenarbeit*		
*Ein mäßig intensives Ausdauertraining erhöht die Atemfrequenz. Ein intensives Ausdauertraining erhöht auch die Herzfrequenz und führt zum Schwitzen. Jede Woche sollten mindestens 150 Minuten mäßig intensives oder 75 Minuten intensives Ausdauertraining oder eine beliebige Kombination davon durchgeführt werden. Intensives Training ist doppelt so effektiv! Zusätzlich zum Ausdauertraining sollten noch mindestens 2 Trainingseinheiten pro Woche zur Verbesserung von Kraft, Beweglichkeit, Gleichgewicht und Koordination durchgeführt werden*. *In den folgenden Empfehlungen meint der Begriff „körperliche Aktivität“ immer auch den etwas genaueren Begriff „Training“*		
**Was sagen die Empfehlungen** *Die EULAR Bewegungsempfehlungen zu körperlicher Aktivität sind gegliedert in 4 übergeordnete Prinzipien und 10 Empfehlungen. Die übergeordneten Prinzipien betonen, dass* *1) körperliche Aktivität Teil eines gesunden Lebensstils ist und die Lebensqualität verbessern kann,* *2) körperliche Aktivität für Menschen mit RA, SpA, Hüft- und Kniegelenkarthrose gesundheitliche Vorteile bringt,* *3) die Empfehlungen zu körperlicher Aktivität für Gesunde auch für Menschen mit RA, SpA, Hüft- und Kniegelenkarthrose wirksam und sicher angewendet werden können,* *4) vor Beginn eines Trainingsprogramms der Bewegungsplan mit professioneller Unterstützung erarbeitet werden sollte und dabei persönliche Vorlieben, Bedürfnisse und Fähigkeiten berücksichtigt werden sollten*		
**Aussagekraft der Empfehlungen** Die Empfehlungen basieren auf vorhandenen wissenschaftlichen Erkenntnissen und Expertenmeinungen. Je mehr Sterne eine Empfehlung hat, desto stärker ist die Aussagekraft und desto wichtiger ist es, diese umzusetzen. Empfehlungen mit 1 oder 2 Sternen basieren auf Expertenmeinungen und auf weniger beweiskräftigen Studien. Empfehlungen mit 1 oder 2 Sternen können jedoch genauso wichtig sein wie diejenigen mit 3 oder 4 Sternen, die durch beweiskräftige Studien belegt sind. Das Fehlen von Beweisen für die Wirksamkeit bedeutet nicht, dass eine Empfehlung nicht trotzdem anwendbar ist		
**Empfehlungen**	Aussagekraft	Grad der Zustimmung (NRS 0–10), durch die Vertreter*innen der Patientenorganisationen (*n* = 5, [83 %]), Mittelwert (SD) Median (Spannweite)
**1) Die Förderung von körperlicher Aktivität sollte ein wesentlicher Bestandteil der Versorgung von Menschen mit RA, SpA, Hüft- und Kniegelenkarthrose während des gesamten Erkrankungsverlaufs sein** Wenn Sie RA, SpA, Hüft- und Kniegelenkarthrose haben, ist es vorteilhaft und ungefährlich, körperlich aktiv zu sein oder die körperliche Aktivität zu steigern. Angesichts des Risikos einer Herz-Kreislauf-Erkrankung für Menschen mit RA oder SpA ist es besonders wichtig, intensives Ausdauertraining durchzuführen. Körperliche Aktivität kann auch die Muskelkraft und Beweglichkeit verbessern sowie Krankheitssymptome lindern. Ihr Behandlungsteam sollte mit Ihnen zusammenarbeiten und Sie in Ihrer körperlichen Aktivität unterstützen	****	10 (0,0) 10 (10–10)
**2) Das Behandlungsteam sollte Verantwortung für die Förderung von körperlicher Aktivität übernehmen und zusammenarbeiten, um angemessene Maßnahmen sicherzustellen** Ihr Behandlungsteam sollte Sie dazu ermutigen, Ihre körperliche Aktivität zu steigern oder aufrechtzuerhalten. Ihr Behandlungsteam sollte miteinander kooperieren und Sie bei Bedarf auch an Spezialisten überweisen, beispielsweise an eine/n Physiotherapeuten/in	*	10 (0,0) 10 (10–10)
**3) Maßnahmen zu körperlicher Aktivität sollten von Fachpersonal durchgeführt werden, das über die notwendige Kompetenz verfügt** Ihr Behandlungsteam sollte die Prinzipien der körperlichen Aktivität verstehen und sie kompetent anwenden. Sie sollten zudem über Kenntnisse bezüglich RA, SpA, Hüft- und Kniegelenkarthrose verfügen	*	9,40 (1,34) 10 (7–10)
**4) Das Behandlungsteam sollte Art, Intensität, Häufigkeit und Dauer der aktuellen körperlichen Aktivität überprüfen und Ziele für Verbesserungen ermitteln** Es gibt 4 Bereiche von körperlicher Aktivität: Ausdauer, Muskelkraft, Beweglichkeit sowie Koordination/Gleichgewicht. Ihr Behandlungsteam stellt Ihnen möglicherweise Fragen, um herauszufinden, welche Arten von körperlicher Aktivität Sie ausführen, und um Sie zu beraten und gemeinsam mit Ihnen Ziele zu vereinbaren	**	8,40 (2,07) 9 (5–10)
**5) Allgemeine oder krankheitsspezifische Hinderungsgründe in Bezug auf die körperliche Aktivität sollten erfasst werden** Es kann allgemeine oder krankheitsspezifische Hinderungsgründe dafür geben, dass Sie bestimmte Arten von körperlicher Aktivität nicht ausführen sollen. Ihr Behandlungsteam wird Sie unter Berücksichtigung der allgemeinen Empfehlungen in Ihrem Land beraten	*	9,60 (0,89) 10 (8–10)
**6) Jede Maßnahme zu körperlicher Aktivität sollte klare personalisierte Ziele haben, welche regelmäßig überprüft werden** Mit Tests zur Erfassung der körperlichen Leistungsfähigkeit, Fragebögen oder Selbstbeobachtung kann der Fortschritt Ihrer körperlichen Aktivität überprüft werden. Möglicherweise ist es hilfreich für Sie, Ihre Fortschritte selbst mit einem elektronischen Gerät wie einem Schrittzähler oder über eine App auf Ihrem Mobiltelefon zu verfolgen	*	7,60 (1,67) 8 (5–9)
**7) Hindernde und unterstützende Faktoren in Bezug auf körperliche Aktivität sollten bei jedem Menschen mit RA, SpA, Hüft- und Kniegelenkarthrose festgestellt und angesprochen werden. Wichtige Faktoren sind: Kenntnisse, Unterstützung durch das soziale Umfeld, Krankheitssymptome und Selbststeuerung** Es ist wichtig, dass Sie Ihre Krankheit verstehen und wissen, wie Sie sicher trainieren können. Sprechen Sie mit Ihrem Behandlungsteam, wenn Sie sich Sorgen machen, dass sich körperliche Aktivität auf Ihre Schmerzen auswirken könnte, oder wenn Sie Angst vor Schüben oder Schädigungen haben. Ihr Behandlungsteam informiert Sie darüber, wie sich Bewegung auf Ihre Krankheitssymptome oder die Kontrolle der Krankheit auswirken kann. Das Behandlungsteam wird mit Ihnen außerdem über das richtige Training, die Einnahme von Schmerzmitteln vor dem Training, die Verwendung von Selbstmotivationstechniken und ein unterstützendes soziales Umfeld sprechen	**	9,99 (2,24) 10 (5–10)
**8) Individuelle Anpassungen an die allgemeinen Empfehlungen zur körperlichen Aktivität sollten auf der Auswertung körperlicher, sozialer und psychologischer Faktoren wie Müdigkeit, Schmerzen, Depressionen und der Krankheitsaktivität beruhen** Viele Menschen mit RA, SpA, Hüft- und Kniegelenkarthrose können die Empfehlungen zu körperlicher Aktivität für Gesunde erreichen. Es kann sich jedoch zeigen, dass der Trainingsplan an den Gesundheitszustand, beispielsweise bei akuten Schmerzen oder lokalen Gelenkentzündungen, angepasst werden sollte	*	9,80 (0,45) 10 (8–10)
**9) Pläne zur Durchführung der körperlichen Aktivität sollten Techniken zur Verhaltensänderung wie Selbstbeobachtung, Zielsetzung, Handlungsplanung, Rückmeldung und Problemlösung umfassen** Um körperliche Aktivität in Ihren Alltag zu integrieren, müssen Sie eventuell Ihren Lebensstil ändern. Wichtige Prinzipien der Verhaltensänderung sind Selbstbeobachtung, Zielsetzung und Handlungsplanung, um die persönlichen Ziele zu erreichen. Ihr Behandlungsteam sollte Ihnen gegebenenfalls bei der Planung behilflich sein, Rückmeldung geben und Sie bei der Lösung von Problemen in Bezug auf Ihre körperliche Aktivität unterstützen	****	8,60 (0,89) 8 (8–10)
10) Die Art der körperlichen Aktivität sollte den persönlichen Vorlieben des/der Patient*in entsprechen Jede Person hat bestimmte Vorlieben, um ein ausreichendes Maß an körperlicher Aktivität während der Woche zu erreichen. Geplante körperliche Aktivitäten können mit oder ohne Anleitung, einzeln oder in Gruppen durchgeführt werden, persönlich vor Ort oder auch online. Was für Sie am besten ist, hängt von Ihren individuellen Vorlieben ab. Es kann auch hilfreich sein, an Wiederholungsmaßnahmen zur Auffrischung des Wissens teilzunehmen. Diese können z. B. auch durch einen persönlichen Telefonanruf oder mithilfe eines tragbaren elektronischen Gerätes (z. B. Schrittzähler) erfolgen	*	9,00 (1,73) 10 (6–10)

## Diskussion

Die laienverständliche deutschsprachige Version der EULAR Bewegungsempfehlungen für Menschen mit RA, SpA und HKA entstand durch einen breit abgestützten und sorgfältig durchgeführten Übersetzungs- und Validierungsprozess. Die hohe Zustimmung zu den einzelnen Empfehlungen legen nahe, dass eine in allen deutschsprachigen Ländern verständliche und akzeptierte Version vorliegt.

Am Übersetzungsprozess nahmen die Vertreter*innen von Patientenorganisationen aktiv teil, und es ist ihnen zu verdanken, dass sich die Version von der professionellen Übersetzung zu einer präfinalen Version in allgemeinverständlicherer Sprache entwickelte. In diesem Prozess wurde um einzelne Worte gerungen, und es zeigte sich eindrücklich, wie unterschiedlich der Gebrauch von spezifischen Wörtern und deren Bedeutung in den 3 deutschsprachigen Ländern ist. Die kognitiven Interviews führten zu weiteren sprachlichen Präzisierungen und damit zur besseren Verständlichkeit. Es gab aber keine systematisch unterschiedlichen Rückmeldungen aus den 3 Ländern.

Der Übersetzungs- und Validierungsprozess wurde gemäß den Leitlinien für Best-Practice von interkulturellen Umfragen gemacht mit professioneller Übersetzung und Expertendiskussion (in diesem Fall Direktbetroffenen), aber ohne Rückübersetzung. Tatsächlich werden Rückübersetzungen immer noch angewendet bei interkulturellen Adaptationen von Fragebogen oder Empfehlungen, aber deren Mehrwert wurde nie belegt und ist zunehmend umstritten [13]. Im Gegensatz dazu brauchen Expertenübersetzer keine Rückübersetzung, weil sie über adäquate Fertigkeiten zur linguistischen Problemidentifikation und -lösung verfügen.

Die hohen Zustimmungen zu den EULAR Bewegungsempfehlungen belegen die gute Verständlichkeit und Akzeptanz, und sie zeigen auch den Stellenwert von körperlicher Aktivität bei den Betroffenen. Gesundheitsdienstleister*innen können die uneingeschränkte Zustimmung zu den Empfehlungen 1 (*körperliche Aktivität als wesentlicher Bestandteil*) und 2 (*Verantwortung übernehmen und zusammenarbeiten*) durchaus als Aufforderung verstehen, die körperliche Aktivität ihrer Patient*innen (vermehrt) zu unterstützen. Körperliche Aktivität sollte Betroffenen evidenzbasiert und genügend hoch dosiert angeboten werden. In der klinischen Praxis lässt sich häufig beobachten, dass Trainingsangebote für Menschen mit rheumatischen Erkrankungen unterdosiert sind, insbesondere in Bezug auf die Intensität von Ausdauer- oder Krafttraining. Ein wichtiger Grund dafür scheinen mangelnde Kenntnisse der Evidenz für die Wirksamkeit und Sicherheit von körperlicher Aktivität bei Menschen mit rheumatischen Erkrankungen zu sein. Es hält sich weiter die Befürchtung, mit höherer Intensität mehr zu schaden als zu nützen [12]. Ärzt*innen sollten das Thema körperliche Aktivität mit ihren Patient*innen besprechen, sie motivieren und in ein passendes Angebot überweisen. Speziell die nichtärztlichen Gesundheitsberufe sollten hier verstärkt eingebunden werden, nicht nur für die Durchführung, sondern auch für die Koordination von und Beratung zu Bewegungsangeboten. Die Motivation und Absicht, sich zu bewegen, wird stark davon beeinflusst, wie wichtig die Ärzteschaft das Thema körperliche Aktivität nimmt [14]. Zu Empfehlung 2 wurde allerdings von Interviewpartnern angemerkt, dass auch die Menschen mit rheumatischen Erkrankungen selbst Verantwortung für ihre körperliche Aktivität übernehmen müssen. Interessanterweise wurde die Empfehlung 3 (*kompetente Fachpersonen*) nicht von allen Betroffenen mit der maximalen Bewertung bewertet. Möglicherweise war das so, weil erlebte persönliche Unterstützung ein starker Unterstützungsfaktor ist, in der Empfehlung 3 aber nicht erwähnt wird [15]. Überraschenderweise wurden die beiden Empfehlungen 4 (*körperliche Aktivität regelmäßig überprüfen*) und 6 (*personalisierte Ziele*) mit Zustimmungen zwischen 5 und 10 respektive 5 und 9 am tiefsten bewertet, aber die Empfehlung 8 (*individuelle Anpassungen*) hatte hohe Zustimmung. Das sind wichtige Hinweise für die Gesundheitsdienstleister*innen, nämlich das Ziel der Evaluationen zu erklären, vorhandene Testergebnisse zu erläutern und darauf abgestützte individualisierte Interventionen vorzuschlagen bzw. durchzuführen. Sehr hohe Zustimmung erhielt auch die Empfehlung 7 (*Barrieren und Förderfaktoren*), allerdings mit Einzelbewertungen von 5 bis 10, wohingegen die Empfehlung 8 (*Verhaltensänderungstechniken verwenden*) eine der mit weniger als 9 bewerteten Empfehlungen war trotz der Evidenz für den Nutzen von Verhaltensänderungstechniken. Die Empfehlung 10 (*körperliche Aktivität gemäß persönlichen Vorlieben*), wurde zwischen 6 und 10 bewertet, was etwas überrascht, da mit Empfehlung 10 den individuellen Vorlieben ja explizit viel Raum gelassen wird.

### Limitationen

Die im Übersetzungsprozess involvierten Vertreter*Innen von Patientenorganisationen waren nicht repräsentativ für die Population von Menschen mit RA, SpA und HKA. Eine Vertreterin konnte nicht anwesend sein und gab vorgängig zum Treffen schriftliche Rückmeldung. Dasselbe gilt auch für die Teilnehmenden an den Interviews, die abhängig von ihrer Verfügbarkeit ausgewählt waren. Die Zustimmung zu den EULAR Empfehlungen und die berechneten durchschnittlichen Übereinstimmungen basieren auf nur 5 Betroffenen, eine Person nahm nicht teil.

## Ausblick

Die deutschsprachige laienverständliche Version der EULAR Empfehlungen zur körperlichen Aktivität soll nun verbreitet und konkret umgesetzt werden. Dabei spielen die Patientenorganisationen eine zentrale Rolle. Die EULAR Empfehlungen zeigen Betroffenen, wie wichtig körperliche Aktivität für ihr Krankheitsmanagement ist und was sie von ihrem Behandlungsteam diesbezüglich erwarten dürfen und sollen. Die Implementierung muss mit zielgruppenspezifischen Strategien unterstützt werden. Der durchgeführte Übersetzungs- und Validierungsprozess kann auch als Modell für weitere Übersetzungen dienen.

## Fazit für die Praxis

Die laienverständliche deutschsprachige Version der EULAR Bewegungsempfehlungen für Menschen mit entzündlich-rheumatischen und degenerativen Erkrankungen unterstreicht die Wichtigkeit von körperlicher Aktivität in der Versorgung von Betroffenen.

- Die Verschreibung von körperlicher Aktivität für Menschen mit rheumatoider Arthritis (RA), Spondyloarthritis (SpA), Hüft- und Kniegelenkarthrose (HKA) ist während des gesamten Erkrankungsverlaufs angebracht.
- Kompetente Fachpersonen führen evidenzbasierte Maßnahmen zu körperlicher Aktivität durch. Diese beinhalten die Evaluation der körperlichen Fitness und personalisierte Ziele und sind angepasst an die individuellen Bedürfnisse und Präferenzen der Betroffenen.
- Fachpersonen verstehen körperliche Aktivität als Teil eines gesunden Lebensstils. Sie evaluieren deshalb die vorhandenen hindernden und fördernden Faktoren und unterstützen die langfristige Integration in den Alltag mit Verhaltensänderungstechniken.
